# Relationships between the degrees of freedom in the affine Gaussian derivative model for visual receptive fields and 2-D affine image transformations with application to covariance properties of simple cells in the primary visual cortex

**DOI:** 10.1007/s00422-025-01014-4

**Published:** 2025-06-18

**Authors:** Tony Lindeberg

**Affiliations:** Computational Brain Science Lab, Division of Computational Science and Technology, KTH Royal Institute of Technology, SE-100 44, Stockholm, Sweden

**Keywords:** Receptive field, Image transformations, Affine, Covariance, Gaussian derivative, Simple cell

## Abstract

When observing the surface patterns of objects delimited by smooth surfaces, the projections of the surface patterns to the image domain will be subject to substantial variabilities, as induced by variabilities in the geometric viewing conditions, and as generated by either monocular or binocular imaging conditions, or by relative motions between the object and the observer over time. To first order of approximation, the image deformations of such projected surface patterns can be modelled as local linearizations in terms of local 2-D spatial affine transformations. This paper presents a theoretical analysis of relationships between the degrees of freedom in 2-D spatial affine image transformations and the degrees of freedom in the affine Gaussian derivative model for visual receptive fields. For this purpose, we first describe a canonical decomposition of 2-D affine transformations on a product form, closely related to a singular value decomposition, while in closed form, and which reveals the degrees of freedom in terms of (i) uniform scaling transformations, (ii) an overall amount of global rotation, (iii) a complementary non-uniform scaling transformation and (iv) a relative normalization to a preferred symmetry orientation in the image domain. Then, we show how these degrees of freedom relate to the degrees of freedom in the affine Gaussian derivative model. Finally, we use these theoretical results to consider whether we could regard the biological receptive fields in the primary visual cortex of higher mammals as being able to span the degrees of freedom of 2-D spatial affine transformations, based on interpretations of existing neurophysiological experimental results.

## Introduction

**Fig. 1:**
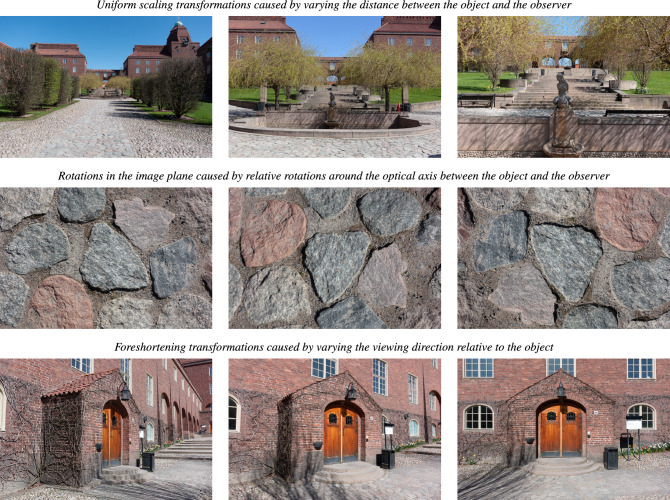
*Basic types of variabilities in natural image data as caused by geometric image transformations:* **(top row)** Uniform scaling transformations caused by varying the distance between the object and the observer. **(middle row)** Rotations in the image domain caused by relative rotations between the object and the observer around the optical axis. **(bottom row)** Foreshortening transformations that lead to non-uniform scaling transformations with the tilt direction (the projection of the surface normal to the image plane) as a preferred orientation in the image plane. Notably, despite these variabilities in the image domain, our perceptual vision system is able to perceive the objects in the world as stable, although the receptive fields, which operate on the set of image values over local regions in image space, will then be exposed to input information that may differ significantly between different views of the same object. In this work, we explore the consequences of using covariance properties of the visual receptive fields as a fundamental constraint, to make it possible to compare and relate the receptive field responses from different views, with *a priori* possible unknown geometric image transformations between such views

When viewing a 3-D object in the environment from different distances and different viewing directions, the projected 2-D images on either the retina or the camera sensor will be subject to a substantial variability, as caused by the variability in the viewing conditions (see Fig. 1 for illustrations).

To first order of approximation, by approximating the perspective mappings from a smooth local surface patch on the object to any two different perspective images by local linearizations (first-order derivatives), the resulting variability of resulting image data from such multi-view observations of the same surface patch can be modelled in terms of local affine transformations of the form

1 $$\begin{aligned} x' = \mathcal{A} \, x. \end{aligned}$$

For simplicity, we have here discarded a possible complementary variability with respect to an added translation vector *b* in the image domain of the form $x' = \mathcal{A} \, x + b$, by without essential loss of generality assuming that all the multi-view observations of the same surface patch are focused on the same viewing point *P* on the 3-D object. The origins of the coordinate systems in the two image domains are also assumed to correspond to the projections of that same fixation point.

A non-trivial, while sometimes overlooked, aspect of visual perception is that we perceive an object in the world as the same, although the 2-D perspective projections of such an object can differ substantially, depending on the viewing distance and the viewing direction of the observer.

A conceptual question that one could then ask is if the ability of perceptual system to derive seemingly stable representations of external objects in the 3-D environment would lead to constraints regarding the variabilities in the shapes of the receptive fields at the earliest levels in the visual processing hierarchy. In a theoretically motivated and axiomatically determined normative theory of visual receptive fields (Lindeberg 2013a, 2021), idealized models for the receptive fields of simple cells in the primary visual cortex (V1) have specifically been derived. These receptive field models lead to provable covariance properties under spatial affine transformations, and regarding extensions to time-dependent spatio-temporal image data also provable covariance properties under Galilean transformations, to handle the variabilities in the relative motion between the objects in the world and the observer. See Lindeberg (2023, 2025c) for more in-depth treatments regarding the importance of covariance properties for visual receptive fields under geometric image transformations.

**Fig. 2:**
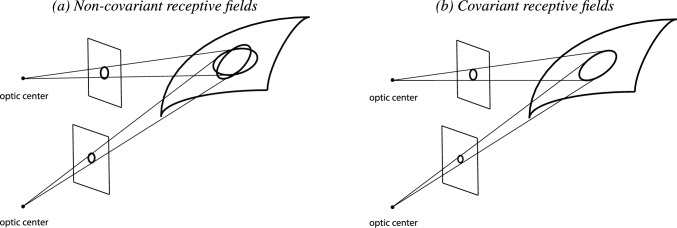
*Geometric illustration of the motivation for the underlying hypothesis concerning affine covariant receptive fields in biological vision*. The importance of the notion of covariance for the receptive fields under geometric image transformations originates from the difference in the backprojected receptive fields that will result, if the receptive fields are not covariant. (a) In the left figure, which shows backprojections of non-covariant receptive fields to the tangent plane of a local surface patch, it is clear that largely different support regions in the tangent plane to the surface will affect the receptive field responses for observations of the same surface patch from different viewing directions. (b) In the right figure, which shows backprojections of covariant receptive fields, for which the parameters of the receptive fields have been additionally matched, such that the backprojections of the receptive fields for the two different observations are equal, the contributions from the different image points within the spatial support regions of the visual receptive fields will contribute in a similar manner to the receptive field responses, which implies that the mismatch source of error to cues about properties of the environment will, to first order of approximation, be completely eliminated. (Figures adapted from Lindeberg (2023) with permission (OpenAccess)

From a computational viewpoint, if we regard the earliest layers in the visual perception system as a computational engine, that infers successively more complex cues about the structure of the environment from the image measurements that reach the retina. Then, covariance properties of visual receptive fields do from a theoretical perspective constitute a highly useful notion. Specifically, if we design a computational algorithm for computing the local surface orientation of a smooth surface patch from binocular cues. Then, compared to not basing the computations on covariant spatial receptive fields, the use of provably affine-covariant visual receptive fields can improve the accuracy by an order of magnitude in estimates of the local surface orientation (see Tables 1–4 in Lindeberg and Gårding (1997)). This is achieved by eliminating a source of error corresponding to a mismatch between the backprojected receptive fields to the tangent plane of the surface, from the two different perspective views, see Figure 2 for an illustration.

Given these theoretically motivated considerations, which have also been empirically tested in computer vision algorithms, one could then raise the question whether the biological vision systems in higher mammals could be regarded as obeying covariance properties under affine spatial transformations, and thus spanning a variability over the degrees of freedom of 2-D spatial affine transformations.

The subject of this paper is to address this issue, by:

- First theoretically describing the different degrees of freedom in spatial affine transformations, in terms of a canonical parameterization, based on a closed-form factorization of the affine transformation matrix $\mathcal{A}$ very closely related to a singular value decomposition.
- Then, relating these degrees of freedom to the degrees of freedom in the parameters of the idealized receptive models of simple cells in the primary visual cortex, in terms of the generalized Gaussian derivative theory for visual receptive fields.
- Finally, relating these results to existing evidence regarding variabilities in the shapes of biological receptive fields established from neurophysiological measurements.

Specifically, we will after an initial overview of related work in Section 2, in Section 3 describe a decomposition of 2-D spatial affine image transformations, based on a closed-form decomposition of the affine transformation matrix in a way very closely related to a singular value decomposition. The added value of this parameterization is that it reveals the degrees of freedom of 2-D spatial affine image transformations in a very geometric manner.

Then, after reviewing the covariance properties of the affine Gaussian derivative model for visual receptive fields in Section 4, we will in Section 5 explicitly describe the degrees of freedom in the affine Gaussian derivative model for visual receptive fields.

Based on this theoretical basis, we will then in Section 6 relate the degrees of freedom in the affine Gaussian derivative model to the degrees of freedom revealed by our proposed decomposition of 2-D affine transformations. This is achieved in terms of explicit mappings between the parameters in our decomposition of 2-D spatial affine transformations and the parameters in the affine Gaussian derivative model. These connections will then be used in Section 7 for addressing the question of whether we can regard the receptive fields in the primary visual cortex as being able to span the degrees of freedom of 2-D affine transformations.

Finally, Section 8 gives a summary of the main results, with suggestions to further neurophysiological studies to firmly determine to what extent the presented biological hypotheses hold in the primary visual cortex, and then also for what species, as well as outlooks concerning further implications of the presented theoretical results.

The presented results are aimed at providing a theoretical understanding of basic covariance properties for the earliest layers of receptive fields in computational vision, as well as for generating hypotheses about the computational functions in the earliest levels of visual processing in biological vision.

A main contribution of this study is that it provides a theoretical framework for how the variability in the spatial receptive field shapes in higher mammals can be accounted for based on symmetry properties of the environment. In particular, the theoretical relations to be derived reveal the practical usefulness for the visual system to explicitly handle geometric image transformations from the 3-D environment to the 2-D visual patterns registered in the earliest layers of the visual hierarchy.

More specifically, if the vision system expands the receptive field shapes over the degrees of freedom of the geometric image transformations. Then, the visual system will have the possibility to compute explicitly readable signals to the higher cortices, that are suitable for a variety of different imaging conditions. Thereby, the expanded image representation can be used for extracting invariant features from the objects that are projected to the retina.

In this way, the visual system can meet the huge variability in spatial or spatio-temporal image structures, that are generated from different possible imaging conditions of any object in the environment by a corresponding variability in the receptive field shapes in the primary visual cortex. By that, the visual system maintains a notion of identity between measurements of the same object performed under different geometric configurations between the object and the observer.

An important consequence of the covariance property of receptive fields, used as a theoretical foundation in the following treatment, is that it makes it possible for receptive field responses to be matched between different viewing conditions. By the notion of geometric covariance, the visual system will be able to meet the variability in image structures generated by geometric image transformations by a corresponding variability in receptive field shapes. This implies that the receptive field responses will, to first order of approximation, be the same under the geometric image transformations. In this way, the visual system achieves a constancy of the representation, given that the parameters of receptive fields can be matched to the geometric image transformations.

Thus, in summary, the proposed theory predicts a set of variabilities in receptive field shapes as induced by corresponding variabilities in image structures, and as generated by the variabilities of geometric image transformations.

## Relations to previous work

Characterizing the functions of the receptive fields in the early visual pathway in terms of computational models constitutes a main topic in the task of understanding the functionalities in the primary visual cortex.

With regard to the task of characterizing the functionality of simple cells, neurophysiological recordings of receptive field profiles of simple cells in the primary visual cortex have been performed by, among others, DeAngelis et al. (1995); DeAngelis and Anzai (2004); Ringach (2002, 2004); Conway and Livingstone (2006); Johnson et al. (2008); Ghodrati et al. (2017); Walker et al. (2019); De and Horwitz (2021).

The receptive field shapes of simple cells have, in turn, been modelled mathematically in terms of, most commonly, either Gabor filters by Marcelja (1980); Jones and Palmer (1987a, 1987b); Porat and Zeevi (1988); Ringach (2002, 2004), or Gaussian derivatives by Koenderink (1984); Koenderink and van Doorn (1987, 1992); Young (1987); Young et al. (2001); Young and Lesperance (2001) and Lindeberg (2013a, 2021). See specifically Figures 16 and 17 in Lindeberg (2021) for examples of how spatial receptive fields of simple cells in the primary visual cortex can be modelled in terms of affine Gaussian derivatives.

Functional modelling of different processes in biological vision has also specifically been performed in terms of Gaussian derivatives by Lowe (2000); May and Georgeson (2007); Hesse and Georgeson (2005); Georgeson et al. (2007); Wallis and Georgeson (2009); Hansen and Neumann (2008); Wang and Spratling (2016) and Pei et al. (2016).

With regard to spatial scaling transformations of the image data that is registered by a visual observer, at a higher level of abstraction, evidence for processing over multiple scales, with scale invariance constituting a basic functional property in biological vision, have been presented by Biederman and Cooper (1992); Logothetis et al. (1995); Ito et al. (1995); Furmanski and Engel (2000); Hung et al. (2005); Wiskott (2006); Isik et al. (2013); Murty and Arun (2017); Benvenuti et al. (2018) and Han et al. (2020).

Studies of the geometric relationships between pairwise binocular views have been presented by Koenderink and van Doorn (1976); Jones and Malik (1992); Gårding and Lindeberg (1994); Mitiche and Létang (1998); Aczél et al. (1999); Uka and DeAngelis (2002); Hansard and Horaud (2008) and Turski (2016, 2024), and more general studies of the image geometry between multiple views by Hartley and Zisserman (2004) and Faugeras (1993).

Concerning invariances of image features to geometric image transformations, invariant representations have been modelled in terms of group theory by Poggio and Anselmi (2016). An overview of computational methods to achieve invariance to scaling transformations and locally linearized perspective transformations for monocular cues, as well as locally linearized projections between projective pairwise views, within a larger framework that also involves invariances to Galilean transformations in joint space-time, has been given in Lindeberg (2013b). Specifically, it is proposed in Lindeberg (2013a, 2013b, 2021) how covariance properties of the receptive fields in the earliest layers of the visual hierarchy make it possible to compute invariant image features at the higher levels, to, for example, support invariant object recognition.

In view of such a computational view to geometric invariance and covariance properties, one may therefore ask if the receptive fields in the primary visual cortex would be covariant under the basic classes of geometric image transformations, as proposed in Lindeberg (2023).

The subject of this paper is to address this issue with regard to the special case of spatial affine image transformations, and specifically based on a canonical decomposition of such 2-D spatial affine transformations. This decomposition is in terms of a product form of (i) a uniform scaling transformation, (ii) pure rotations, (iii) a non-uniform scaling transformation, and (iv) a normalization of the non-uniform scaling transformation to a preferred symmetry orientation in the image domain.

An earlier study of parameterizations of affine transformations, which we will build upon in this work, was presented in Lindeberg (1995). In this paper, we extend that parameterization by first of all providing an explicit derivation in Appendix A.1, which was not presented in the original paper because of space limitations. Then, we analyse the properties of this decomposition for four basic classes of primitive affine transformations in Appendix A.2. Additionally, we give a more general treatment regarding special cases for the parameters in the affine transformation model, which thereby increases the domain of applicability of the proposed parameterization to more general families of affine transformation matrices, that are reasonably near the unit matrix multiplied by a scalar scaling factor.

Then, we will relate the degrees of freedom obtained from this decomposition arising parameterization of 2-D spatial affine transformations to the variabilities in the shapes of the receptive fields in the primary visual cortex.

**Fig. 3:**
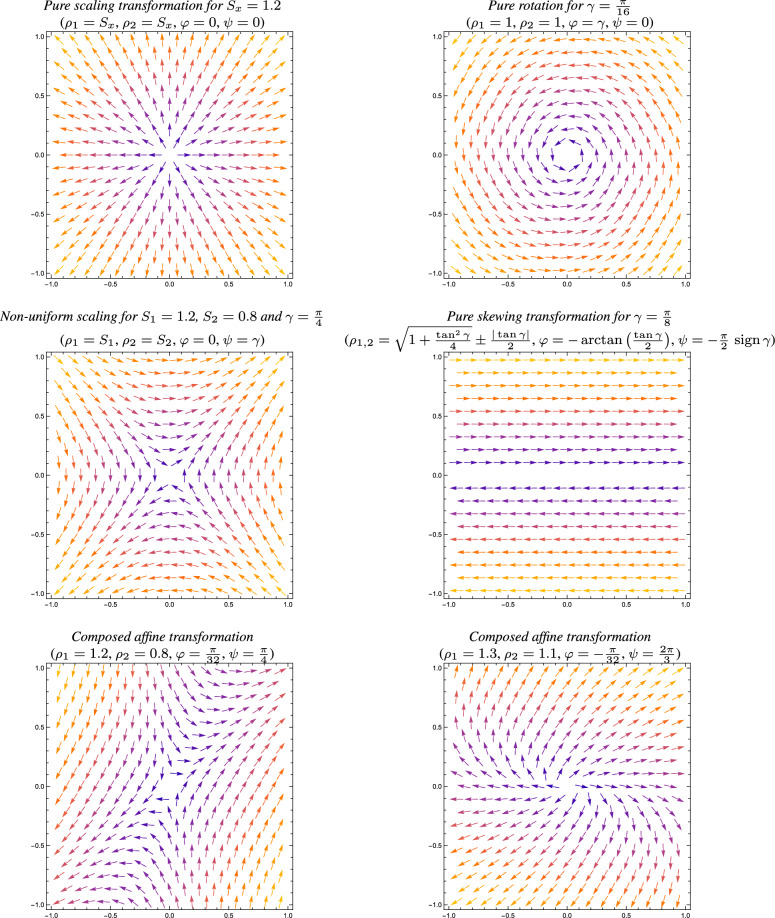
Visualizations of deformation fields $\varDelta x = x' - x = \mathcal{A} \, x - x = (\mathcal{A} - \mathcal{I}) \, x$ **(top and middle rows)** for four basic types of primitive affine transformations and **(bottom row)** two composed affine image transformations, with the directions of the deformation vectors $\varDelta x$ shown as arrows of unit length, and with the magnitude of the deformation coded in colour, with blue corresponding to low magnitudes and yellow to higher magnitudes. For illustration, also the parameters ($\rho _1$, $\rho _2$, $\varphi$, $\psi$) in the proposed decomposition of the affine transformation matrix according to (18) are shown, based on the detailed analysis of these four special cases of primitive affine image transformations in Appendix A.2, with specifically the pure scaling transformation matrix according to (102), the pure rotation matrix according to (113), the non-uniform scaling transformation according to (124) and the skewing transformation according to (137). (**Horizontal axes:** spatial coordinate $x_1$. **Vertical axes:** spatial coordinate $x_2$.)

## The degrees of freedom in 2-D spatial affine transformations

Let us parameterize the affine transformation matrix $\mathcal{A}$ in the affine transformation (1) as

2 $$\begin{aligned} \mathcal{A} = \left( \begin{array}{cc} a_{11} & a_{12} \\ a_{21} & a_{22} \end{array} \right) \end{aligned}$$

and parameterize the image coordinates as $x = (x_1, x_2)^T$ and $x' = (x'_1, x'_2)^T$. Here, we are specifically interested in the special case when the affine transformation matrix $\mathcal{A}$ is reasonably close to the unit matrix $\mathcal{I}$ multiplied by some scalar scaling factor.

Following Section 3 in (Lindeberg 1995), let us next define the following descriptors from the elements $a_{ij}$ of $\mathcal{A}$:

3 $$\begin{aligned} T&= \frac{a_{11} + a_{22}}{2},\end{aligned}$$

4 $$\begin{aligned} A&= \frac{a_{21} - a_{12}}{2},\end{aligned}$$

5 $$\begin{aligned} C&= \frac{a_{11} - a_{22}}{2},\end{aligned}$$

6 $$\begin{aligned} S&= \frac{a_{12} + a_{21}}{2}. \end{aligned}$$

Let us also define the following derived descriptors

7 $$\begin{aligned} P&= \sqrt{T^2 + A^2},\end{aligned}$$

8 $$\begin{aligned} Q&= \sqrt{C^2 + S^2}. \end{aligned}$$

Then, according to the treatment in Appendix A.1, specifically Equations (88)–(89), it follows that, if we decompose the affine transformation matrix $\mathcal{A}$ into a modified singular value decomposition of the form

9 $$\begin{aligned} \mathcal{A} = \mathcal{R}_{\alpha } \, \operatorname {diag}(\rho _1, \rho _2) \, \mathcal{R}_{\beta }^T, \end{aligned}$$

where

10 $$\begin{aligned} \mathcal{R}_{\alpha } = \left( \begin{array}{cc} \cos \alpha & - \sin \alpha \\ \sin \alpha & \cos \alpha \end{array} \right) \end{aligned}$$

and

11 $$\begin{aligned} \mathcal{R}_{\beta } = \left( \begin{array}{cc} \cos \beta & - \sin \beta \\ \sin \beta & \cos \beta \end{array} \right) \end{aligned}$$

are enforced to be pure rotation matrices, then the diagonal entries $\rho _1$ and $\rho _2$ in the diagonal matrix $\operatorname {diag}(\rho _1, \rho _2)$ in the decomposition (9) are given by

12 $$\begin{aligned} \rho _1&= P + Q,\end{aligned}$$

13 $$\begin{aligned} \rho _2&= P - Q. \end{aligned}$$

Due to our assumption of the affine transformation being reasonably close to the unit matrix $\mathcal{I}$ multiplied by some scalar scaling factor, we will for the purpose of this treatment specifically assume that $\rho _1> \rho _2 > 0$. Then, the decomposition (9) also constitutes a genuine singular value decomposition^1^ of the affine transformation matrix $\mathcal{A}$, with the notable distinction that the matrices $\mathcal{R}_{\alpha }$ and $\mathcal{R}_{\beta }$ are here guaranteed to be actual rotation matrices.

If we furthermore define the following angles

14 $$\begin{aligned} \tan \varphi&= \frac{A}{T},\end{aligned}$$

15 $$\begin{aligned} \tan \psi&= \frac{S}{C}, \end{aligned}$$

as well as the corresponding rotation matrices

16 $$\begin{aligned} \mathcal{R}_{\frac{\varphi }{2}} = \left( \begin{array}{cc} \cos \frac{\varphi }{2} & - \sin \frac{\varphi }{2} \\ \sin \frac{\varphi }{2} & \cos \frac{\varphi }{2} \end{array} \right) \end{aligned}$$

and

17 $$\begin{aligned} \mathcal{R}_{\frac{\psi }{2}} = \left( \begin{array}{cc} \cos \frac{\psi }{2} & - \sin \frac{\psi }{2} \\ \sin \frac{\psi }{2} & \cos \frac{\psi }{2} \end{array} \right) , \end{aligned}$$

then it can be shown (see Appendix A.1 specifically Equations (100) and (101)) that the singular value decomposition of the affine transformation matrix $\mathcal{A}$, can be factorized on the form

18 $$\begin{aligned} \mathcal{A}&= \mathcal{R}_{\frac{\psi }{2}} \, \mathcal{R}_{\frac{\varphi }{2}} \operatorname {diag}(\rho _1, \rho _2) \, \mathcal{R}_{\frac{\varphi }{2}} \, \mathcal{R}_{-\frac{\psi }{2}} \nonumber \\&= \sqrt{\rho _1 \, \rho _2} \,\, \mathcal{R}_{\frac{\psi }{2}} \, \mathcal{R}_{\frac{\varphi }{2}} \operatorname {diag}\left( \sqrt{\frac{\rho _1}{\rho _2}}, \sqrt{\frac{\rho _2}{\rho _1}}\right) \, \mathcal{R}_{\frac{\varphi }{2}} \, \mathcal{R}_{-\frac{\psi }{2}}, \end{aligned}$$

where

- $\rho _1$ and $\rho _2$ are the singular values of $\mathcal{A}$,
- the factor
  19 $$\begin{aligned} S = \sqrt{\rho _1 \, \rho _2} \end{aligned}$$
  corresponds to an overall spatial scaling transformation,
- the diagonal matrix
  20 $$\begin{aligned} \mathcal{D} = \operatorname {diag}\left( \sqrt{\frac{\rho _1}{\rho _2}}, \sqrt{\frac{\rho _2}{\rho _1}}\right) \end{aligned}$$
  describes a non-uniform stretching transformation, corresponding to a relative spatial stretching factor of
  21 $$\begin{aligned} \lambda = \frac{\rho _1}{\rho _2}, \end{aligned}$$
- $\mathcal{R}_{\frac{\psi }{2}}$ and $\mathcal{R}_{\frac{\varphi }{2}}$ are rotation matrices with rotation angles $\frac{\psi }{2}$ and $\frac{\varphi }{2}$, respectively.

In terms of overall effects of the affine transformation, the interpretations of the two rotation angles $\varphi$ and $\psi$ are then, specifically, that:

- $\varphi = \frac{\varphi }{2} + \frac{\varphi }{2}$ describes the overall total amount of spatial rotation,
- $\frac{\psi }{2}$ represents the orientation of a local symmetry axis for the non-isotropic part of the affine transformation, *e.g.* the pure stretching transformation.

The top and the middle rows in Figure 3 show examples of spatial deformation fields for four basic classes of primitive affine transformations, with the parameters for each one of the different special cases determined according to the analysis of these four special cases in Appendix A.2. The bottom row in Figure 3 does additionally show examples of composed affine deformation fields, as specified from the parameters $(\rho _1, \rho _2, \varphi , \psi )$ in the proposed decomposition of affine transformation matrices according to (18).

**Fig. 4:**
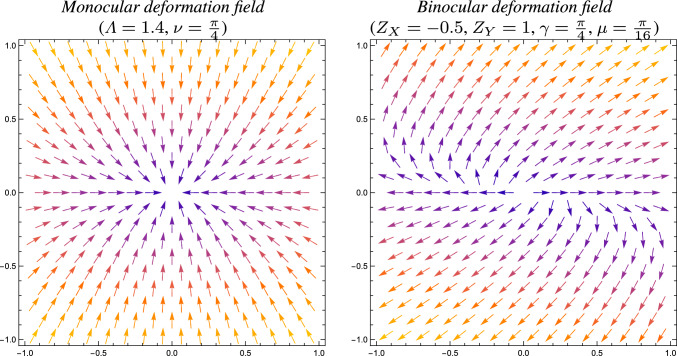
*Visualizations of deformation fields* $\varDelta x = x' - x = \mathcal{A} \, x - x = (\mathcal{A} - \mathcal{I}) \, x$ from locally linearized monocular and binocular perspective or projective projection models. **(left)** A monocular deformation field as arising from a locally linearized perspective projection according to (22) of a slanted surface with slant angle $\nu$ under variations of the distance $\Lambda$ between the object and the observer. **(right)** A binocular deformation field as arising from a locally linearized projective mapping between the tangent planes of two smooth surfaces according to (23), for specific values of the depth gradient $\nabla Z = (Z_X, Z_Y)$, the gaze angle $\gamma$ between the frontal direction and direction to the fixation point as well as the opening angle $2\mu$ between the viewing directions of the two observers. (**Horizontal axes:** spatial coordinate $x_1$. **Vertical axes:** spatial coordinate $x_2$)

### Geometric interpretations of affine image deformations

As previously mentioned, let us consider surface patterns of possibly moving smooth objects in the world that are projected to the image plane, by either (i) the perspective mapping to a monocular image, (ii) the projective mapping between two binocular views, or (iii) the temporally integrated optic flow fields obtained by observing moving objects between adjacent time moments. Then, the resulting image transformations or deformation fields can to first order of approximation be modelled as local affine transformations.

For this purpose, the decomposition in (18) constitutes a geometrically very natural way to parameterize the degrees of freedom in such local affine transformations of the perspective/projective mappings. Specifically, these geometric image transformations determine the variabilities of the image structures, that a visual system is exposed to, when observing sufficiently smooth surface patterns in the environment from different viewing directions relative to the objects in the environment.

#### Monocular locally linearized perspective projection

To make such relations more explicit, consider, for example, the monocular perspective projection of a smooth local surface patch onto a spherical camera. Let us specifically consider two local coordinate systems, with

- the first local coordinate system being in the tangent plane of the surface patch, with the second local coordinate direction aligned with the tilt^2^ direction of the surface and
- the second local coordinate system being in the tangent plane of the spherical camera, with the second local coordinate direction aligned with the backprojected tilt direction onto the resulting image plane parallel to the tangent plane of the surface.

Then, the first-order locally linearized mapping from the tangent plane of the surface to the tangent plane on the spherical camera is given by (see Gårding and Lindeberg (1996) Equation (28), although here reformulated with somewhat different conventions and notation)

22 $$\begin{aligned} \mathcal{A}_{\text{ mono }} = \frac{1}{\Lambda } \left( \begin{array}{cc} 1 & 0\\ 0 & \cos \nu \end{array} \right) , \end{aligned}$$

where

- $\Lambda$ is the distance between the observed point on the surface of the object and the observer, and
- $\nu$ is the slant angle, that is the angle between the surface normal to the surface of the object and the viewing direction.

Figure 4(left) shows a schematic illustration of such a deformation field, as arising from the appearance of deformations of a local surface pattern on a planar surface to the image plane, as we by the local linearization disregard higher-order non-linear components in the perspective mapping.

#### Binocular locally linearized projective projection

In the binocular case, consider two eyes or cameras that observe a smooth local surface patch from a cyclopean observer. This means that locus of the cyclopean observer is on the midpoint between the two optical centers of the cameras on a circle through the two optical centers and the observed point on the surface, see Figure 1 in Gårding and Lindeberg (1994) for an illustration.

Let $2 \mu$ denote the angle between the viewing directions from the two eyes or cameras to the fixation point, and let $\gamma$ denote the gaze angle between the frontal direction and the direction to the fixation point. Furthermore, let $Z_X$ and $Z_Y$ represent the components of the depth gradient, with *Z* denoting the depth, and *X* and *Y* being the world coordinates parallel to the directions of the image coordinates $x_1$ and $x_2$, respectively. Then, the disparity gradient from the left image to the right image for a calibrated visual observer is given by (see Gårding and Lindeberg (1994) Equation (3))

23 $$\begin{aligned} \mathcal{A}_{\text{ bino }}&= \left( \begin{array}{cc} 1 + h_1 & h_2 \\ v_1 & 1 + v_2 \end{array} \right) \nonumber \\&= \frac{\cos (\gamma - \mu )}{\cos (\gamma + \mu )} \left( \begin{array}{cc} \frac{\cos \mu + Z_X \, \sin \mu }{\cos \mu - Z_X \, \sin \mu } & \frac{Z_Y \, \sin 2 \mu }{\cos \mu - Z_X \, \sin \mu } \\ 0 & 1 \end{array} \right) , \end{aligned}$$

where $\nabla h = (h_1, h_2)^T$ denotes the horizontal disparity gradient and $\nabla v = (v_1, v_2)^T$ denotes the vertical disparity gradient.

Figure 4(right) shows a schematic example of such a disparity field, as arising from a local linearization between corresponding points under the projective mapping between the two image domains (seen from the two views) for specific values of the geometric parameters $\mu$, $\gamma$ and $(Z_X, Z_Y)$.

## Covariance properties of affine Gaussian derivative based receptive fields under spatial affine transformations

To characterize the effects that affine image transformations have on receptive field responses, let us next consider receptive fields according to the generalized Gaussian derivative model for visual receptive fields (Lindeberg 2021). With this regard, let us specifically only consider the effects in relation to the pure spatial and spatio-temporal smoothing transformations applied to static image data or video data, respectively.

### Affine covariance for purely spatial receptive fields

For a purely spatial receptive field applied on pure spatial image data, the influence of a spatial affine transformation on a receptive field response can be described as the convolution of any input image *f*(*x*) with an affine Gaussian kernel $g(x;\; \varSigma )$ (see Lindeberg (1993) Equation (15.17))

24 $$\begin{aligned} L(x;\; \varSigma ) = (g(\cdot ;\; \varSigma ) * f(\cdot ))(x;\; \varSigma ) \end{aligned}$$

where

25 $$\begin{aligned} g(x;\; \varSigma ) = \frac{1}{2 \pi \sqrt{\det \varSigma }} \, e^{-x^T \varSigma ^{-1} x/2}. \end{aligned}$$

Then, it can be shown that, under an affine transformation of the image domain $f'(x') = f(x)$ for $x' = \mathcal{A} \, x$ according to (1), the corresponding purely spatial scale-space representation according to (24) over the transformed domain

26 $$\begin{aligned} L'(x';\; \varSigma ') = (g(\cdot ;\; \varSigma ') * f'(\cdot ))(x';\; \varSigma ') \end{aligned}$$

is related to the scale-space representation (24) over the original domain according to

27 $$\begin{aligned} L'(x';\; \varSigma ') = L(x;\; \varSigma ), \end{aligned}$$

provided that the spatial covariance matrices $\varSigma '$ and $\varSigma$ in the two images domains (before and after the geometric image transformation) are related according to (see Equations (29) and (30) in Lindeberg and Gårding (1997))

28 $$\begin{aligned} \varSigma ' = \mathcal{A} \, \varSigma \, \mathcal{A}^T. \end{aligned}$$

Given these raw purely smoothed components of the receptive field responses, corresponding receptive field responses in terms of spatial derivatives over the two image domains (before and after the geometric image transformation), smoothed by affine Gaussian kernels with matching spatial covariance matrices $\varSigma$ and $\varSigma '$, can, in turn, be related according to

29 $$\begin{aligned} \nabla _{x'} = \mathcal{A}^{-T} \, \nabla _{x}. \end{aligned}$$

These relationships, thus, show that, provided that spatial covariance matrices are related in an appropriate way, according to an actual affine image transformation, it is then possible to match the receptive field responses perfectly between the two image domains. For such matching to be possible in an actual situation, the actual value of the spatial covariance matrices, and hence the shapes of the corresponding affine Gaussian derivative based receptive fields, must thereby be adapted to the actual affine transformation. Specifically, if the input stimuli involve a variability over a certain subspace or subspace of the degrees of freedom of the affine transformations. Then, the family of receptive field shapes must also comprise a variability over a that corresponding subspace or subdomain of the parameter space of the affine Gaussian derivative based receptive fields, in order for it to be possible to perfectly match the outputs from the affine Gaussian derivative kernels between the two image domains.

### Affine covariance for joint spatio-temporal receptive fields

A similar analysis can also be performed for joint spatio-temporal receptive fields according to the idealized model (see Lindeberg (2021) Equation (24))

30 $$\begin{aligned} T(x, t;\; \varSigma , \tau , v) = g(x - v t;\; \varSigma ) \, h(t;\; \tau ) \end{aligned}$$

where $\tau$ denotes the temporal scale in units of the temporal variance $\tau = \sigma _t^2$ of the temporal smoothing kernel $h(t;\; \tau )$ and $v = (v_1, v_2)$ denotes an image velocity parameter.

Given two video sequences *f*(*x*, *t*) and $f'(x', t')$, that are related according to a pure spatial affine transformation

31 $$\begin{aligned} x' = \mathcal{A} \, x, \quad \quad \text{ and } \quad \quad t' = t, \end{aligned}$$

let

32 $$\begin{aligned}&L(x, t;\; \varSigma , \tau , v) \nonumber \\&\quad = (T(\cdot , \cdot ;\; \varSigma , \tau , v) * f(\cdot , \cdot ))(x, t;\; \varSigma , \tau , v), \end{aligned}$$

33 $$\begin{aligned}&L'(x', t';\; \varSigma ', \tau ', v') \nonumber \\&\quad = (T(\cdot , \cdot ;\; \varSigma ', \tau ', v') * f'(\cdot , \cdot ))(x', t';\; \varSigma ', \tau ', v'), \end{aligned}$$

denote the spatio-temporal smoothed scale-space representations in the two spatio-temporal image domains. Then, these joint spatio-temporal scale-space representations are related according to (see Lindeberg (2023) Equation (54))

34 $$\begin{aligned} L'(x', t';\; \varSigma ', \tau ', v') = L(x, t;\; \varSigma , \tau , v), \end{aligned}$$

provided that the spatial covariance matrices $\varSigma$ and $\varSigma '$ are related according to

35 $$\begin{aligned} \varSigma ' = \mathcal{A} \, \varSigma \, \mathcal{A}^T, \end{aligned}$$

as well as provided that the other receptive field parameters are equal, that is $\tau ' = \tau$ and $v' = v'$. Thus, this result shows that also joint spatio-temporal receptive field responses can be made to perfectly match each other under spatial affine transformations, provided that the spatial shapes of the joint spatio-temporal receptive fields are appropriately adapted with respect to the actual spatial affine transformation.

**Fig. 5:**
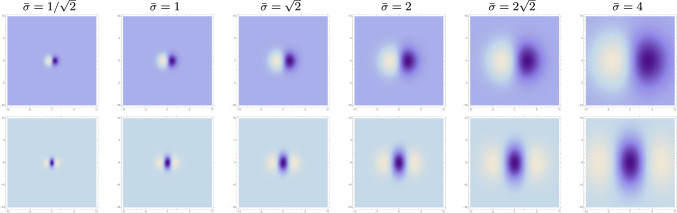
*Variability in the size *of affine Gaussian derivative receptive fields (for $\sigma _1 = \sigma _2$ and image orientation $\varphi = 0$), with the overall size $\bar{\sigma } = \sqrt{\sigma _1 \sigma _2}$ increasing from $1/\sqrt{2}$ to 4 according to a logarithmic distribution, from left to right. (top row) First-order directional derivatives of affine Gaussian kernels according to (43). (bottom row) Second-order directional derivatives of affine Gaussian kernels according to (44). (**Horizontal axes:** image coordinate $x_1 \in [-10, 10]$. **Vertical axes:** image coordinate $x_2 \in [-10, 10]$.)

**Fig. 6:**
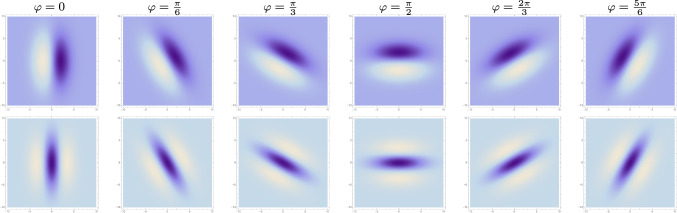
*Variability in the orientation* of affine Gaussian derivative receptive fields (for $\sigma _1 = 2$ and $\sigma _2 = 4$), with the orientation angle $\varphi$ increasing from left to right. (top row) First-order directional derivatives of affine Gaussian kernels according to (43). (bottom row) Second-order directional derivatives of affine Gaussian kernels according to (44). (**Horizontal axes:** image coordinate $x_1 \in [-10, 10]$. **Vertical axes:** image coordinate $x_2 \in [-10, 10]$.)

**Fig. 7:**
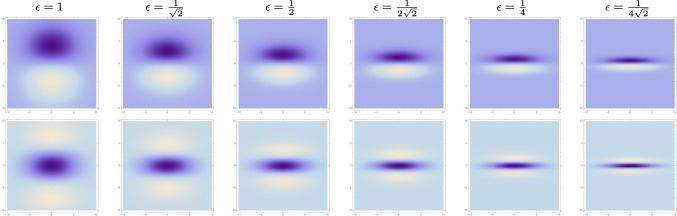
Variability in the eccentricity of affine Gaussian derivative receptive fields (for image orientation $\varphi = \frac{\pi }{2}$), with the eccentricity $\epsilon = \sigma _1/\sigma _2$ decreasing from 1 to according to a logarithmic distribution, from left to right, with $\sigma _2$ kept constant. (top row) First-order directional derivatives of affine Gaussian kernels according to (43). (bottom row) Second-order directional derivatives of affine Gaussian kernels according to (44). (**Horizontal axes:** image coordinate $x_1 \in [-10, 10]$. **Vertical axes:** image coordinate $x_2 \in [-10, 10]$.)

## Degrees of freedom in the affine Gaussian derivative model for visual receptive fields

The spatial covariance matrix

36 $$\begin{aligned} \varSigma = \left( \begin{array}{cc} C_{11} & C_{12} \\ C_{12} & C_{22} \end{array} \right) \end{aligned}$$

in the affine Gaussian derivative model for visual receptive fields can be parameterized on the form

37 $$\begin{aligned} C_{11}&= \sigma _1^2 \, \cos ^2 \varphi + \sigma _2^2 \, \sin ^2 \varphi ,\end{aligned}$$

38 $$\begin{aligned} C_{12}&= (\sigma _1^2 - \sigma _2^2) \cos \varphi \, \sin \varphi ,\end{aligned}$$

39 $$\begin{aligned} C_{22}&= \sigma _1^2 \, \sin ^2 \varphi + \sigma _2^2 \, \cos ^2 \varphi , \end{aligned}$$

where $\lambda _1 = \sigma _1^2$ and $\lambda _2^2= \sigma _2^2$ constitute the eigenvalues of $\varSigma$. Under variabilities of the spatial scale parameters $\sigma _1 > 0$ and $\sigma _2 > 0$, and the spatial orientation $\varphi$, these variabilities span the variability of the shapes of the receptive fields in the receptive field model.

In relation to overall shape properties of the receptive fields, the degrees of freedom in this parameterization have the following interpretation:

- The product
  40 $$\begin{aligned} \bar{\sigma } = \sqrt{\sigma _1 \, \sigma _2} \end{aligned}$$
  describes the overall *spatial size* of the receptive field.
- The ratio
  41 $$\begin{aligned} \epsilon = \frac{\sigma _2}{\sigma _1} \end{aligned}$$
  describes the eccentricity or the *degree of elongation* of the receptive field, in the sense that deviations in this ratio from the rotationally symmetric special case when $\epsilon = 1$ correspond to more elongated or anisotropic receptive fields.
- The angle $\varphi$ in (37)–(39) represents the *spatial orientation* of the receptive field.

Figures 5–7 show illustrations of these variabilities for purely spatial receptive fields, in terms of first- and second-order directional derivatives of affine Gaussian kernels, according to the following idealized models for the receptive fields of simple cells in the primary visual cortex (based on Equation (31) in Lindeberg (2021)):

42 $$\begin{aligned} T_{\text{ simple }}(x_1, x_2;\; \sigma _1, \sigma _2, \varphi , m) = \sigma _1^m \, \partial _{\varphi }^m \, g(x_1, x_2;\; \varSigma ), \end{aligned}$$

which for first and second orders of spatial differentiation *m* assume the following explicit forms

43 $$\begin{aligned}&T_{\text{ simple }}(x_1, x_2;\; \sigma _1, \sigma _2, \varphi , 1) \nonumber \\&\quad = \sigma _1 \, (\cos (\varphi ) \, \partial _{x_1} + \sin (\varphi ) \, \partial _{x_2}) \, g(x_1, x_2;\; \varSigma ) \nonumber \\&\quad = -\frac{(x_1 \cos (\varphi )+x_2 \sin (\varphi ))}{2 \pi \, \sigma _1^2 \, \sigma _2} \times \nonumber \\&\quad e^{ -\frac{\left( \sigma _1^2+\sigma _2^2\right) \left( x_1^2+x_2^2\right) -(\sigma _1-\sigma _2) (\sigma _1+\sigma _2) (2 x_1 x_2 \sin (2 \varphi )+\cos (2 \varphi ) (x_1-x_2) (x_1+x_2))}{4 \sigma _1^2 \sigma _2^2}} \end{aligned}$$

44 $$\begin{aligned}&T_{\text{ simple }}(x_1, x_2;\; \sigma _1, \sigma _2, \varphi , 2) \nonumber \\&\quad = \sigma _1^2 \, (\cos ^2 (\varphi ) \, \partial _{x_1 x_1} + 2 \cos (\varphi ) \sin (\varphi ) \, \partial _{x_1 x_2} + \sin ^2(\varphi ) \, \partial _{x_2 x_2}) \, \nonumber \\&\quad \quad \quad g(x_1, x_2;\; \varSigma ) \nonumber \\&\quad = \frac{\left( \cos (2 \varphi ) \left( x_1^2-x_2^2\right) +2 x_1 x_2 \sin (2 \varphi )-2 \sigma _1^2+x_1^2+x_2^2\right) }{4 \pi \, \sigma _1^3 \, \sigma _2} \nonumber \\&\quad \times e^{ -\frac{\left( \sigma _1^2+\sigma _2^2\right) \left( x_1^2+x_2^2\right) -(\sigma _1-\sigma _2) (\sigma _1+\sigma _2) (2 x_1 x_2 \sin (2 \varphi )+\cos (2 \varphi ) (x_1-x_2) (x_1+x_2))}{4 \sigma _1^2 \sigma _2^2}}. \end{aligned}$$

Figures 5–7 illustrate the basic variabilities of the shapes of the receptive fields arising in this way according to the affine Gaussian derivative model. A main question addressed in this paper concerns what variabilities in receptive field shapes may be spanned in the primary visual cortex.

## Relationships between the variabilities in affine image transformations and the variabilities in affine Gaussian receptive fields

From a comparison between the degrees of freedom in 2-D spatial affine transformations according to Section 3 with the degrees of freedom in the shapes of the affine Gaussian receptive fields in Section 5, it does specifically hold that:

- Variabilities in the *size* of the overall size receptive field, as represented by the product
  45 $$\begin{aligned} \bar{\sigma } = \sqrt{\sigma _1 \, \sigma _2} \end{aligned}$$
  in (40), span the variability over the overall *spatial scaling factor*
  46 $$\begin{aligned} S = \sqrt{\rho _1 \, \rho _2} \end{aligned}$$
  according to (19) in the affine image transformations.
- Variabilities in the *eccentricity* of the receptive field, as can be described by the ratio
  47 $$\begin{aligned} \epsilon = \frac{\sigma _2}{\sigma _1} \end{aligned}$$
  in (41), do, for a fixed value of the image orientation angle $\varphi$ of the affine Gaussian receptive fields, span the variability in the *ratio between the singular values*
  48 $$\begin{aligned} \lambda = \frac{\rho _1}{\rho _2} \end{aligned}$$
  of the affine transformation, which, in turn, represents the essential variability in the amount of non-uniform spatial stretching in the pure stretching transformation $\mathcal{D}$ according to (20) in the affine image transformations.
- Variabilities in the *orientation angle*
  49 $$\begin{aligned} \varphi \end{aligned}$$
  of the affine Gaussian receptive fields according to (37)–(39) span the variability in the amount of *overall rotation* $\varphi$ of the total rotation component
  50 $$\begin{aligned} \mathcal{R}_{\varphi } = \mathcal{R}_{\frac{\varphi }{2}} \mathcal{R}_{\frac{\varphi }{2}} \end{aligned}$$
  according to (18) in the affine image transformations.

In these respects, there is a one-to-one mapping between three of the degrees of freedom of 2-D spatial affine transformations and the three degrees of freedom in the regular formulation of idealized receptive field models in terms of the regular formulation of directional derivatives of affine Gaussian kernels.

Specifically, if the image data in an imaging situation would be subject to these degrees of freedom in 2-D spatial affine transformations. Then, we can let the affine Gaussian derivative kernels exhibit corresponding variabilities in the shapes of their receptive fields, to be able to match the receptive field responses between the image domains before *vs.* after the affine image deformation.

**Fig. 8:**
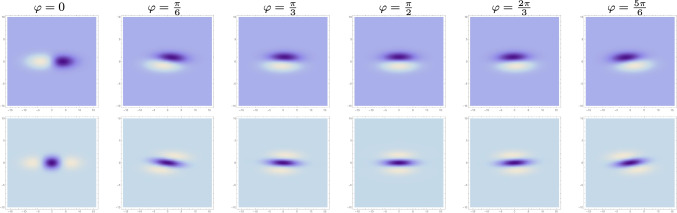
*Variability in the orientation of directional derivatives* of affine Gaussian derivative receptive fields (for $\sigma _1 = 4$ and $\sigma _2 = 1$ and preferred image orientation $\varphi = 0$), with the orientation angle $\varphi$ for the directional derivative operators increasing from left to right. (top row) First-order directional derivatives of affine Gaussian kernels. (bottom row) Second-order directional derivatives of affine Gaussian kernels. (**Horizontal axes:** image coordinate $x_1 \in [-10, 10]$. **Vertical axes:** image coordinate $x_2 \in [-10, 10]$)

### Restricted 3-D variability of regular affine Gaussian derivative kernels

Consider the affine Gaussian derivative model used for formulating the explicit expressions for the idealized models for the receptive fields of simple cells according to Equations (43) and (44), with the directions for which the directional derivatives of the affine Gaussian kernels are computed in directions parallel with the eigendirections of the affine Gaussian kernels. Because the variability of the resulting shapes of affine Gaussian derivative-based receptive fields in three-dimensional, while the variability of the affine image transformations is four-dimensional. Therefore, the variability of the shapes of the receptive fields according that regular affine Gaussian derivative model, in its original formulation, cannot span the full variability of the affine group.

### Possible extensions to a 4-D variability to enable matching of affine Gaussian derivative responses

An operational explanation, why affine covariance cannot be achieved for the regular affine Gaussian derivative kernels, with the orientation for computing the directional derivative of that regular affine Gaussian derivative kernel being required to be aligned with a principal direction of the covariance matrix of the affine Gaussian kernel, can be stated as follows: If the affine image transformation comprises a non-uniform scaling transformation in a direction that not coincides with the principal directions of the spatial covariance matrix in the affine Gaussian smoothing kernel. Then, the transformed receptive field of a regular affine Gaussian derivative kernel, will be no longer within the definition of such a regular affine Gaussian directional derivative kernel.

Instead, to enable full affine covariance, the image orientation of the directional derivative operator in the transformed affine Gaussian derivative kernel must be allowed to be different from the restriction of the image direction for the directional derivative being aligned to either of the principal directions of the affine Gaussian smoothing kernel.

#### Alternative 1: Explicit expansion over the directions of directional derivatives of affine Gaussian kernels

The variability of the receptive fields of receptive fields according to the affine Gaussian derivative model for visual receptive fields can, however, be extended. Consider the orientation angle for which the directional derivatives in the direction $\varphi$ of the affine Gaussian derivative kernels are computed according to (42)

51 $$\begin{aligned} T_{\text{ simple }}(x_1, x_2;\; \sigma _1, \sigma _2, \varphi , m) = \sigma _1^m \, \partial _{\varphi }^m \, g(x_1, x_2;\; \varSigma ). \end{aligned}$$

Let us instead allow the directional derivatives to be computed in directions $\varphi$ in the spatial domain that do not necessarily have to be aligned with either of the eigenvectors of the spatial covariance matrix $\varSigma$, see Figure 8 for examples of such receptive fields for fixed values of the spatial scale parameters $\sigma _1$ and $\sigma _2$. Then, such extended models for the spatial receptive fields, which will span a four-dimensional variability, will allow for expanded covariance properties over the full group of non-singular spatial affine transformations, according to the treatment in (Lindeberg (2025c) Section 3.5).

#### Alternative 2: Expanded matching to linear combinations of affine Gaussian directional derivatives as opposed to restricted matching to just plain receptive field responses

Concerning the above possible extension, it should, however, be noted that due to the definition of directional derivatives, the directional derivative of a 2-D affine Gaussian kernel can be parameterized to the form

52 $$\begin{aligned}&T_{\varphi ^{m_1}\bot \varphi ^{m_2},\text{ norm }}(x_1, x_2;\; \sigma _1, \sigma _2, \varphi , m) \nonumber \\&\quad = \partial _{\varphi ,\text{ norm }}^{m_1} \, \partial _{\bot \varphi , \text{ norm }}^{m_2} \, g(x_1, x_2;\; \varSigma ) \nonumber \\&\quad = \sigma _1^m \, (\cos \varphi \, \partial _{x_1} + \sin \varphi \, \partial _{x_2})^{m_1} \nonumber \\&\quad \phantom {=} \quad \, \sigma _2^{m_2} \, (-\sin \varphi \, \partial _{x_1} + \cos \varphi \, \partial _{x_2})^{m_2} \, g(x_1, x_2;\; \varSigma ), \end{aligned}$$

where $\bot \varphi$ denotes the orthogonal direction to $\varphi$.

Therefore, given a fixed value of the spatial covariance matrix $\varSigma$, it is sufficient to compute the directional derivatives in $m + 1$ sufficiently different directions, in order to then be able to span the space for computing the directional derivative of order $m = m_1 + m_2$ in any other direction, according to

53 $$\begin{aligned}&T_{\varphi ^{m}}(x_1, x_2;\; \sigma _1, \sigma _2, \varphi , m) \nonumber \\&\quad = \sum _{k=1}^M p_k \, T_{\varphi _k^{m}}(x_1, x_2;\; \sigma _1, \sigma _2, \varphi _k, m), \end{aligned}$$

for some $M \ge m + 1$ and some constants $p_k$. For this purpose, the actual values for the parameters $p_k$ can, in turn, be determined by setting up and solving a linear system of equations relations, based on relationships of the form (52), mapped down to relationships in terms of the partial derivatives $\partial _{x_1^i x_2^{m-i}} \, g(x_1, x_2;\; \varSigma )$ as the underlying basis for these relationships for all $j \in [0, m]$.

Thus, from a computational viewpoint, it is not necessary to fully span the variability of this fourth dimension, while nevertheless being able to match the receptive field responses between the two images domains, before and after the geometric image transformation. This can be achieved by simultaneously expanding the matching process to not just match plain receptive field responses between the two image domains, but instead allowing for matching of the receptive field response from the first image domain to a linear combination of receptive field responses over the second image domain of the form

54 $$\begin{aligned}&T_{\varphi ^{m}}(x_1, x_2;\; \sigma _1, \sigma _2, \varphi , m) * f(x_1, x_2) \nonumber \\&\quad = \sum _{k=1}^M q_k \, T_{{\varphi '}_k^{m}}(x'_1, x'_2;\; \sigma '_1, \sigma '_2, \varphi '_k, m) * f'(x'_1, x'_2) \end{aligned}$$

for some constants $q_k$ chosen specific to the relationships between the orientation $\varphi$ in the first domain and the selection of sampled orientations $\varphi '$ in the second domain, as well as also depending on the degrees of freedom of the affine transformation matrix $\mathcal{A}$.

Note, however, that for such a computational structure to work, for directional derivatives of higher orders $m \ge 2$, it is not sufficient to use the directional derivatives along the eigendirections of the spatial covariance matrix $\varSigma$ as the basis. Instead, for directional derivative orders $m \ge 2$, directional derivatives would also have to be computed in directions that are significantly different from the eigendirections of the spatial covariance matrix $\varSigma$. Furthermore, that number of additional required complementary directions for directional derivative computations would be required to increase with the order *m* of spatial differentiation.

## Which degrees of freedom of 2-D spatial affine transformations are spanned by the receptive fields in the primary visual cortex?

Given (i) these theoretical results, and given that (ii) the biological receptive fields corresponding to simple cells in the primary visual cortex, as measured by DeAngelis et al. (1995); DeAngelis and Anzai (2004); Conway and Livingstone (2006) and Johnson et al. (2008), can be qualitatively rather well modelled with spatial components in terms of affine Gaussian derivatives according to the axiomatically derived normative theory for visual receptive fields in Lindeberg (2013a, 2021), one may ask if biological vision has also developed corresponding variabilities in receptive field shapes as would be predicted from the presented theory. Specifically, one may ask if the shapes of the simple cells in the primary visual cortex would have the ability to span the variabilities corresponding to 2-D spatial affine transformations.

As previously stated, a highly useful property of affine covariant receptive fields in computer vision is that they allow for substantially more accurate inference of shape from monocular or binocular cues as opposed to non-covariant receptive fields. See Tables 1–4 in Lindeberg and Gårding (1997) for numerical results of computing estimates of local surface orientation using a procedure that successively updates the shapes of the receptive fields to previous orientation estimates, which then leads to substantially lower errors in the surface orientation estimates after just a few iterations.

As previously argued in Lindeberg (2021) Section 6, it could specifically also constitute an evolutionary advantage for higher species, that rely on visual information as a critical source of information about the environment, to adapt their vision systems to the geometrical properties of the image formation process. Specifically, an expansion of the image data over the degrees of freedom over the parameters of natural image transformations would be consistent with the substantial number of receptive fields in the early visual pathway, with

- about 100 M photoreceptors and 1 M output channels in and from the retina to the lateral geniculate nucleus (LGN),
- about 1 M neurons in the LGN and about 1 M output channels to the primary visual cortex (V1), then
- with about 190 M neurons in V1 and about 37 M output channels from V1,

see Figure 3 in DiCarlo et al. (2012).

The subject of this section is to use the theoretical treatment developed in the previous sections to consider whether we could from such a view regard the receptive fields in the primary visual cortex to exhibit variabilities in their shapes, that could be interpreted as if the receptive fields would have the ability to span the variabilities generated by the family of 2-D spatial affine transformations.

### Variability under uniform scaling transformations

The issue of a possible variability of the receptive fields with respect spatial scaling transformations is special, in the sense that, according to the theory for affine Gaussian receptive fields, the receptive fields at coarser levels of scale can, in principle, be computed from the receptive fields at finer levels of scales.

Consider the semi-group property of the affine Gaussian kernel (see Lindeberg (1993) Equation (15.35))

55 $$\begin{aligned} g(\cdot ;\; \varSigma _1) * g(\cdot ;\; \varSigma _2) = g(\cdot ;\; \varSigma _1 + \varSigma _2), \end{aligned}$$

from which it follows that by combining *e.g* the output from the receptive fields corresponding to first-order spatial derivatives

56 $$\begin{aligned} (\nabla _x \, L)(\cdot ;\; \varSigma _1) = (\nabla _x \, g)(\cdot ;\; \varSigma _1) * f(\cdot ), \end{aligned}$$

where $\nabla _x = (\partial _{x_1}, \partial _{x_2})^T$, over different positions in image space. Due to this semi-group property, we can from the output of such a first layer of visual processing compute the responses of the first-order derivatives at any coarser level of scale

57 $$\begin{aligned} (\nabla _x \, L)(\cdot ;\; \varSigma _2) = (\nabla _x \, g(\cdot ;\; \varSigma _2) ) * f(\cdot ), \end{aligned}$$

by combining the outputs from the first layer with suitable weights, such that the resulting computations implement a convolution operation of the following form

58 $$\begin{aligned} (\nabla _x \, L)(\cdot ;\; \varSigma _2) = g(\cdot ;\; \varSigma _2 - \varSigma _1) * (\nabla _x \, L)(\cdot ;\; \varSigma _1), \end{aligned}$$

provided that the difference between the spatial covariance matrices

59 $$\begin{aligned} \varDelta \varSigma = \varSigma _2 - \varSigma _1 \end{aligned}$$

is a symmetric positive definite matrix.

In fact, concerning receptive fields in the retina, an interpretation of results concerning measurements of receptive fields at different distances from the center of the fovea, in combination with a theoretically principled model of a foveal scale space. This holds if we add a complementary essential requirement of a limited processing capacity in terms of a finite number of neurons. Then, the resulting model is consistent with the interpretation that the minimum size of the receptive fields, at any distance from the center of the fovea, should increase linearly with the distance from the center of the fovea (see Lindeberg (2013a) Section 7).

Thus, irrespective of whether the primary visual cortex would perform an explicit expansion over multiple receptive field sizes over some scale range or not, one could, because of the cascade smoothing property of spatial Gaussian derivative operators, also conceive an alternative possible design strategy for a vision system. According to such an alternative strategy, the vision system could only implement a first layer of visual receptive fields at a finest level of scale, and then computing the representations at coarser scale in an implicit manner, from the finer-scale receptive field responses.

Explicit suggestions for neurophysiological experiments to map the possible variability of receptive field shapes over multiple sizes in the image domain are given in Section 3.2.2 in Lindeberg (2023).

### Variability under rotations in the image plane

**Fig. 9:**
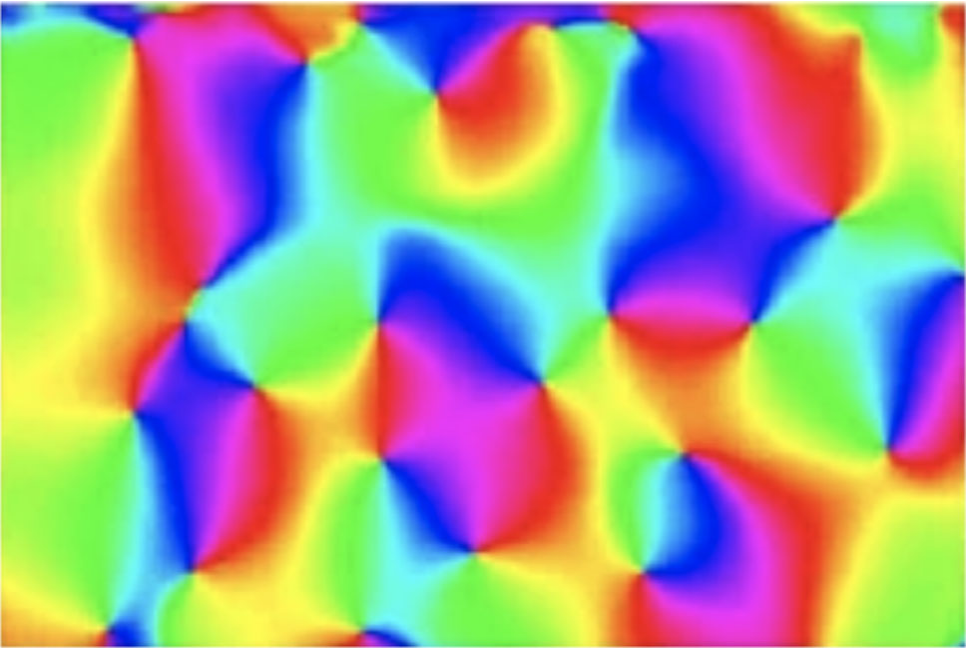
Orientation map in the primary visual cortex of cat, as recorded by Koch et al. (2016) (OpenAccess), and demonstrating that the visual cortex performs an explicit expansion of the receptive field shapes over spatial image orientations, as would be the result of combining the notion of covariance over the subgroup of pure image rotations, and corresponding to a variability over the orientation parameter $\varphi$ in the proposed decomposition (18) of 2-D spatial affine transformations

From the structure of the orientation maps around pinwheels, as pioneered by Bonhoeffer and Grinvald (1991); Blasdel (1992) (see Figure 9), we can from the above theoretical treatment interpret these results as the receptive fields in the visual cortex could be regarded as spanning a variability over the pure image rotation component $\varphi$ in (18) in the affine group.

Furthermore, using a stimulus manifold analysis, Beshkov and Einevoll (2023) show that rotating stimuli in the image domain leads to the generation of circles in the primary visual cortex, also in very good agreement with the assumption of a covariance property in the primary visual cortex over the degree of freedom in affine image transformations corresponding to pure rotations in the image domain.

### Variability under non-uniform scaling transformations?

In Lindeberg (2025a), an in-depth treatment is given concerning whether the receptive fields in the primary visual cortex could be regarded as spanning a variability over the degree of elongation of the receptive fields.

Based on the relationships between the degrees of freedom in 2-D spatial affine transformations and the degrees of freedom in the affine Gaussian derivative model established in Section 6. Then, the possible existence of such a variability, for the receptive fields in the primary visual cortex of higher mammals with a pinwheel structure, would thereby correspond to the family of the receptive fields spanning an expansion over the non-isotropic scaling component $\mathcal{D} = \operatorname {diag}(\sqrt{\frac{\rho _1}{\rho _2}}, \sqrt{\frac{\rho _2}{\rho _1}})$ in (18) of the affine group, that is over the parameter $\lambda = \rho _1/\rho _2$ according to (48) in the proposed decomposition of 2-D spatial affine transformations.

Unfortunately, there does, however, not appear to exist any sufficiently extensive direct measurements of the eccentricity or the degree of elongation for sufficiently large populations of visual neurons to firmly answer this question. In Lindeberg (2025a), an alternative indirect approach has therefore been taken to this topic, by making use of existing biological measurements of orientation selectivity by Nauhaus et al. (2008). They have reported that there is a substantial variability in the orientation selectivity of the visual receptive fields in relation to the pinwheel structure for monkeys and cats. Goris et al. (2015) have also accumulated histograms of the resultant of the orientation selectivity curves for simple cells and complex cells.

In Lindeberg (2025b), an in-depth theoretical analysis is performed of the orientation selectivity properties of idealized models of the receptive fields of simple and complex cells in terms of affine Gaussian derivatives. It is shown that there is a direct connection between the orientation selectivity and the degree of elongation of the receptive fields according to the affine Gaussian derivative model. A complementary in-depth analysis that leads to qualitatively similar results for more developed models of complex cells is given in Lindeberg (2025d). In Lindeberg (2025a), closed-form expressions for the resultant of the orientation selectivity curves are furthermore computed for the idealized model for visual receptive fields based on affine Gaussian derivatives. It is demonstrated that such a closed-form theoretical analysis leads to predictions about histograms of the resultants of orientation selectivity curves for the corresponding idealized models of simple cells that are in very good qualitative agreement with experimentally obtained histograms from biological neurons by Goris et al. (2015).

If we could assume that the idealized generalized affine Gaussian derivative model would constitute a sufficiently accurate model for the receptive fields of the visual neurons in the primary visual cortex, then we would be able to logically infer that there ought to be a variability over the degree of elongation of the receptive fields in the biological neurons. Such logically based modelling arguments are, however, not necessarily guaranteed to hold, if there would be other factors, not incorporated into the idealized models of the receptive fields, that could also affect the orientation selectivity of the receptive fields.

To more firmly determine whether the hypothesis about an expansion over the degree of elongation of the receptive fields would hold for actual biological neurons, a set of more explicit biological hypotheses, with suggestions for complementary quantitative measurements, have therefore been formulated in Sections 3.2–3.3 in Lindeberg (2025a) to answer this question. If the working hypothesis would hold, then the proposed types of neurophysiological measurements could then also characterize how such a possible variability in the degree of elongation of the receptive fields would relate to the pinwheel structures in the orientation maps of the primary visual cortex.

### Possible variability over a fourth degree of freedom?

Concerning a possible additional variability over the remaining fourth degree of freedom of affine transformations, we did in Section 6.2 discuss two ways by which affine Gaussian derivative model with a genuine 3-D variability could be extended to four degrees of freedom.

Notably, in relation to his Gabor modelling of visual receptive fields, Ringach (2002) found that for a majority of the receptive fields that he modelled in that study, he did not find it necessary to add a parameter to vary the orientation of the non-isotropic Gaussian kernel in relation to the orientation of the cosine wave; such a parameter “helped only in a small number of cases” (see Ringach (2002), page 457).

Given that a few receptive fields, hence, obviously have been recorded, for which a Gabor-based modelling of receptive fields would be helped by having the principal axes of an affine Gaussian kernel being not oriented in a similar orientation as the principal axis of the cosine function in the Gabor model, one may ask the following: If instead modelling those receptive fields by directional derivative of affine Gaussian kernels, would such modelling be helped by not having the orientation of the directional derivative operator in the affine Gaussian derivative model being aligned with a principal direction of the affine Gaussian kernel used in that affine Gaussian derivative model?

Given this observation, one may then raise the question of whether it would be possible to accumulate support from neurophysiological experiments for such a variability, which would then constitute support for an expansion of the receptive field shapes over this remaining fourth degree of freedom.

In this context, it is also interesting to note that for some of the receptive field profiles reconstructed by Yazdanbakhsh and Livingstone (2006) (see Figure 6 in that paper) appear to be more similar to first- or second-order directional derivatives of Gaussian kernels in directions different from the principal directions of an affine Gaussian kernel compared to directional derivatives of such kernels in directions that coincide with the principal directions of affine Gaussian kernels. Thus, those reconstructed receptive fields appear to be more similar to members of the receptive field family illustrated in Figure 8 than to the members of the receptive field families illustrated in Figures 5–7.

## Summary and discussion

In this paper, we have given an overall treatment of the variabilities in image structures on the retina or the image plane that are generated by viewing 3-D objects in the environment from different distances and viewing directions, and how the resulting geometric image transformations will interact with the receptive fields in the earliest layers in the visual hierarchy. For convenience of mathematical analysis, we have throughout modelled these geometric image transformations in terms of spatial affine transformations, corresponding to the derivative of the perspective mapping from smooth local surface patches in the world to the retinal surface, or corresponding to the derivative of the projective mappings between pairwise views of the same surface patch from different viewing directions and distances.

For the purpose of establishing an identity relation between receptive field responses computed from different views of the same local surface patch, we have investigated the consequences of the assumption that the receptive field family should be covariant under the locally linearized perspective or projective transformations, thereby assuming that the receptive fields should be covariant under spatial affine transformations. As a main working hypothesis, we have thus investigated the consequences of this assumption in the respect of the simple cells in the primary visual cortex being covariant to spatial affine transformations. This covariance property would then mean that the visual receptive fields in the primary visual cortex would meet the variabilities in spatial image structures as generated by the geometric image transformations by a corresponding variability in the receptive field shapes, in order to enable matching of receptive field responses under different viewing conditions of the same object. A manifest implication of that covariance property would then be that the receptive field shapes in the primary visual cortex would span a similar variability as generated by the variability of spatial affine transformations, and thereby span the degrees of freedom of spatial affine transformations.

The main subject of this paper has been to develop a theoretical foundation for investigating the consequences of this way of reasoning, by first relating the degrees of freedom of spatial affine transformations to the degrees of freedom in our highly idealized affine Gaussian derivative model for visual receptive fields. Then, we have considered for which matches of degrees of freedom in the geometric image transformations to the corresponding degrees of freedom in the theoretical receptive field model, we could identify corresponding variabilities in the receptive field shapes of simple cells in the primary visual cortex.

A longer-term underlying motivation to this work is that the presented theoretical foundation could then be used as a basis for more detailed neurophysiological or psychophysical studies, concerning to what extent biological vision for different types species may have developed receptive field structures that have similar properties as the predictions obtained from the highly idealized theory.

More technically, we have, after describing contextual relations to previous work in Section 2, first in Section 3 performed an in-depth characterization of the degrees of freedom in 2-D spatial affine transformations, based on a decomposition of the affine transformation matrix to a product form very closely related to a singular value decomposition, although on closed form, and with the pre-multiplication and post-multiplication matrices *U* and *V* in a regular singular value decomposition here required to be pure rotation matrices.

For our target application, where we consider affine transformation matrices reasonably close to a unit matrix multiplied by a positive scaling factor, such a product form does indeed guarantee positive diagonal entries in the diagonal matrix of the proposed matrix decomposition. In Appendix A.1, we have specifically shown how the parameters $(\rho _1, \rho _2, \varphi , \psi )$ of the proposed matrix decomposition (18) can be determined from the parameters $a_{ij}$ of the affine transformation matrix $\mathcal{A}$, which in these ways extend the earlier treatment of this concept in Lindeberg (1995).

Then, we have, after an overview of the covariance properties of the affine Gaussian derivative model in Section 4, in Section 5 analyzed the degrees of freedom in the affine Gaussian derivative model, to in Section 6 relate the degrees of freedom of the affine Gaussian derivative model to the degrees of freedom in 2-D spatial affine transformations:

- For the uniform spatial scaling factor $S = \sqrt{\rho _1 \, \rho _2}$ in a spatial affine transformation decomposed according to (19), there is a direct mapping to the the square root of the product of the standard deviations $\bar{\sigma } = \sqrt{\sigma _1 \, \sigma _2}$ according to (45) of the Gaussian kernel in the principal directions of the spatial covariance matrix $\varSigma$.
- For the ratio between the singular values $\lambda = \rho _1/\rho _2$ in the affine decomposition according to (48), there is a direct (inverse) relationship to the eccentricity $\epsilon = \sigma _2/\sigma _1$ according to (47) in the receptive field model based on affine Gaussian derivatives.
- For the total amount of rotation $\mathcal{R}_{\varphi } = \mathcal{R}_{\frac{\varphi }{2}} \mathcal{R}_{\frac{\varphi }{2}}$ according to (50) in the proposed matrix decomposition (18) , there is a direct mapping to variabilities in the orientation $\varphi$ of the affine Gaussian receptive fields according to (42), provided that the spatial covariance matrix covaries with the angle $\varphi$ according to (37)–(39).

Due to the fact that the original formulation of idealized models of simple cells $T_{\text{ simple }}(x_1, x_2;\; \sigma _1, \sigma _2, \varphi , m)$ according to the most strict formulation according to (42), with the directional derivative operator parallel to either of the eigendirections of the spatial covariance matrix $\varSigma$, only represents a three-dimensional variability, while the variability of affine transformation matrices $\mathcal{A}$ is four-dimensional, the restriction of idealized simple cells to the form (42) cannot span the full variability of 2-D spatial affine transformations.

To enable matching of receptive field responses under the full variability of 4-D spatial transformations, we have therefore in Section 6.2 considered two types of extensions of the original model, to enable prefect matching of receptive field responses under general 2-D spatial affine transformations:

- Either by extending the variability of the directions of the directional derivatives to a full variability over image orientations in relation to the eigendirections of the spatial covariance matrix $\varSigma$, which, however, then would lead to a highly redundant representation, since the directional derivatives in different directions are related in terms of linear combinations.
- Or indirectly representing just the subspace of such a full variability with a set of at least $m + 1$ sampled and sufficiently different image orientations $\varphi _k$ in relation to the eigendirections of the spatial covariance matrix, and then performing the matching to linear combinations of receptive field responses within that subspace, as opposed to perfect pairwise matching of receptive field responses between the two image domains that are related by a 2-D spatial affine transformations.

In these ways, we have described how the idealized affine Gaussian derivative model allows for explicit matching of first of all spatial receptive field responses under general 2-D spatial affine transformations, based on the covariance properties of the spatial affine Gaussian derivative model according to the treatment in Section 4.1. With extension of the spatial affine covariance properties to spatio-temporal affine covariance according to Section 4.2, the proposed model does additionally allow for matching of spatio-temporal receptive field responses computed based on the idealized model for spatio-temporal receptive fields according to (30), when complemented by spatial and temporal differentiation according to the theory presented in Lindeberg (2023, 2025c).

Finally, we have in Section 7 considered if we could from the theory predict if the receptive fields of the simple cells and complex cells would have the ability to span similar types of variabilities in receptive field shapes as inferred from the presented theory:

- From existing results, by combining the spans over the degrees of freedom corresponding to uniform scaling transformations and rotations, it appears clear that we could regard the primary visual cortex to be able to be covariant under similarity transformations, that is to combinations of uniform scaling transformations and rotations.
- If we additionally would interpret the potential support in Lindeberg (2025a) for the hypothesis that the receptive field shapes would additionally be expanded over different degrees of elongations, as predicted from the variabilities of the degree of orientation selectivity established from neurophysiological recordings by Nauhaus et al. (2008); Goris et al. (2015). Then, it seems natural to also predict that the receptive fields in the primary visual cortex could exhibit properties as would be obtained if the primary visual cortex would have the ability to be covariant also over a significant span over the group of 2-D spatial affine transformations, that is also to image transformations involving non-uniform scaling transformations, as naturally arise from variations of the slant angle, when observing smooth local surface patches in the world from different viewing directions.

To firmly establish if these predictions, based on our highly idealized theory for visual receptive fields, would hold in reality in the primary visual cortex of higher species, further biological experiments would, however, be extremely valuable, for which explicitly testable experimental hypotheses concerning specific subgroups of 2-D spatial affine transformations have been outlined in Section 3.2.1 in Lindeberg (2023) and Sections 3.2–3.3 in Lindeberg (2025a).

### Outlook

If it could be established that the receptive field shapes in the primary visual cortex would exhibit a variability corresponding to the degrees of freedom in 2-D spatial affine transformations. Then, such a result would constitute partial support for the working hypothesis that the receptive fields in the primary visual cortex of higher mammals could be regarded as an affine covariant family of basis functions, that performs an expansion of the incoming image data over the degrees of freedom of 2-D spatial image transformations. A topic of particular interest could also be to investigate if the presence of such covariance properties would be different for different species, and, if so, concerning what species.

The presented theory and predictions have immediate implications for electrophysiological characterization of receptive fields as well as for psychophysical studies of vision.

One main line of possible future research would concern reconstructing a large set of neurophysiologically recorded receptive field shapes in the primary visual cortex of higher mammals. Specifically, to map the variabilities in the parameters of the receptive fields (their spatial extent in the principal directions, their orientation, as well as the possible deviation between the direction of a directional derivative operator from the principal directions of the underlying spatial smoothing kernel). In relation to the more detailed proposals concerning such neurophysiological measurements outlined in some additional respects^3^ in Lindeberg (2023, 2025a), one may specifically investigate if there would be fundamental differences between lower and higher species, as well as regarding species that either have or not have a pinwheel structure. The developed notion of affine image transformations in relation to receptive field measurements could possibly also be combined with the approach in Yazdanbakhsh and Livingstone (2006) to extract second-order receptive fields, to electrophysiologically characterize the aperture problem, and infer the connectivities in the pathway from the retina, upward to the lateral geniculate nucleus and the primary visual cortex.

Concerning psychophysical experiments, another interesting direction could be to try to explore the influence of evolutionarily- or learning-based priors in the visual system that enable biological vision to very quickly solve visual tasks that from a purely mathematical modelling perspective could be regarded as ambiguous or ill-posed, given that the visual system faces the task of inferring 3-D cues about the environment from lower-dimensional 2-D image data. Could it be established that a pre-wiring of the visual system to achieve constancy of object properties in the environment under variations in the viewing conditions could be characterized in terms of psychophysical measurements, to in turn relate to local affine transformations of image patterns and covariance or invariance properties of the underlying receptive fields under such geometric image transformations, as addressed in this treatment?

For example, regarding visual illusions, the accordion grating illusion, where a non-uniform deformation field perpendicular to the lines is perceived when viewing a set of parallel lines under variations of the distance to the observer, would according to its theoretical explanation (Yazdanbakhsh and Gori 2011) not exist without the existence and influence of local affine image transformations. Additionally, in the rotating tilted lines illusion, where a non-zero radial motion is perceived when viewing a static stimulus consisting of a set of gradually rotated line segments that form a circle at a larger scale, with a geometric interpretation corresponding to the interior of a 3-D tube, can according to its theoretical explanation (Yazdanbakhsh and Gori 2008) be used to estimate the minimum receptive field size as function of the distance from the centre of the visual field.

Irrespective of such possible biological implications, the theoretical analysis of the degrees of freedom of 2-D spatial affine transformations, with its relations to the degrees of freedom in the affine Gaussian derivative model, is also important for modelling and understanding the computational functions in computer vision systems, that derive information about the environment from dense measurements of image structures or surface patterns from smooth surfaces in the environment.

## Data Availability

No datasets were generated or analysed during the current study.

## Appendix

### Derivation of the decomposition and the parameterization of 2-D spatial affine transformation matrix

#### Basic definitions

Consider a 2-D spatial affine transformation of the form

60 $$\begin{aligned} \mathcal{A} = \left( \begin{array}{cc} a_{11} & a_{12} \\ a_{21} & a_{22} \end{array} \right) \end{aligned}$$

and introduce the following descriptors from the elements $a_{ij}$ of $\mathcal{A}$:

61 $$\begin{aligned} T&= \frac{a_{11} + a_{22}}{2}, \end{aligned}$$

62 $$\begin{aligned} A&= \frac{a_{21} - a_{12}}{2}, \end{aligned}$$

63 $$\begin{aligned} C&= \frac{a_{11} - a_{22}}{2}, \end{aligned}$$

64 $$\begin{aligned} S&= \frac{a_{12} + a_{21}}{2}. \end{aligned}$$

#### Product decompositions of the affine transformation matrix

Let us next decompose the affine transformation matrix $\mathcal{A}$ according to a singular value decomposition

65 $$\begin{aligned} \mathcal{A} = U \, \varSigma \, V^T, \end{aligned}$$

where *U* and *V* are unitary matrices and $\varSigma$ a diagonal matrix. Let us, however, next rewrite this decomposition as

66 $$\begin{aligned} \mathcal{A} = \mathcal{R}_{\alpha } \, \operatorname {diag}(\rho _1, \rho _2) \, \mathcal{R}_{\beta }^T, \end{aligned}$$

where

67 $$\begin{aligned} \mathcal{R}_{\alpha } = \left( \begin{array}{cc} \cos \alpha & - \sin \alpha \\ \sin \alpha & \cos \alpha \end{array} \right) \end{aligned}$$

and

68 $$\begin{aligned} \mathcal{R}_{\beta } = \left( \begin{array}{cc} \cos \beta & - \sin \beta \\ \sin \beta & \cos \beta \end{array} \right) \end{aligned}$$

are rotation matrices, and $\operatorname {diag}(\rho _1, \rho _2)$ is a diagonal matrix with the elements $\rho _1$ and $\rho _2$.

#### Relations between the parameters of the product decomposition and the TACS descriptors

By expanding the decomposition (66)

69 $$\begin{aligned} \mathcal{A} = \left( \begin{array}{cc} \cos \alpha & - \sin \alpha \\ \sin \alpha & \cos \alpha \end{array} \right) \left( \begin{array}{cc} \rho _1 & 0 \\ 0 & \rho _2 \end{array} \right) \left( \begin{array}{cc} \cos \beta & - \sin \beta \\ \sin \beta & \cos \beta \end{array} \right) ^T \end{aligned}$$

and identifying with the elements $a_{ij}$ in (60) we then obtain

70 $$\begin{aligned}&a_{11} = \rho _1 \, \cos \alpha \, \cos \beta + \rho _2 \, \sin \alpha \, \sin \beta , \end{aligned}$$

71 $$\begin{aligned}&a_{12} = \rho _1 \, \cos \alpha \, \sin \beta - \rho _2 \, \sin \alpha \, \cos \beta , \end{aligned}$$

72 $$\begin{aligned}&a_{21} = \rho _1 \, \sin \alpha \, \cos \beta - \rho _2 \, \cos \alpha \, \sin \beta , \end{aligned}$$

73 $$\begin{aligned}&a_{22} = \rho _1 \, \sin \alpha \, \sin \beta + \rho _2 \, \cos \alpha \, \cos \beta . \end{aligned}$$

By next using the following trigonometric relationships

74 $$\begin{aligned}&\sin (x + y) = \sin x \, \cos y + \cos x \, \sin y, \end{aligned}$$

75 $$\begin{aligned}&\sin (x - y) = \sin x \, \cos y - \cos x \, \sin y, \end{aligned}$$

76 $$\begin{aligned}&\cos (x + y) = \cos x \, \cos y - \sin x \, \sin y, \end{aligned}$$

77 $$\begin{aligned}&\cos (x - y) = \cos x \, \cos y + \sin x \, \sin y, \end{aligned}$$

we then obtain that the explicit expressions for the TACS descriptors according to (61)–(64) are given by

78 $$\begin{aligned} T&= \frac{1}{2} \, (\rho _1 + \rho _2) \, \cos (\alpha - \beta ), \end{aligned}$$

79 $$\begin{aligned} A&= \frac{1}{2} \, (\rho _1 + \rho _2) \, \sin (\alpha - \beta ), \end{aligned}$$

80 $$\begin{aligned} C&= \frac{1}{2} \, (\rho _1 - \rho _2) \, \cos (\alpha + \beta ), \end{aligned}$$

81 $$\begin{aligned} S&= \frac{1}{2} \, (\rho _1 - \rho _2) \, \sin (\alpha + \beta ). \end{aligned}$$

#### Relationships between the singular values $\rho _1$ and $\rho _2$ and the derived PQ descriptors

By further introducing the derived PQ descriptors according to

82 $$\begin{aligned} P&= \sqrt{T^2 + A^2}, \end{aligned}$$

83 $$\begin{aligned} Q&= \sqrt{C^2 + S^2}, \end{aligned}$$

we then obtain that

84 $$\begin{aligned} P&= \frac{1}{2} \, | \rho _1 + \rho _2 |, \end{aligned}$$

85 $$\begin{aligned} Q&= \frac{1}{2} \, | \rho _1 - \rho _2 |. \end{aligned}$$

#### Explicit expressions for the singular values $\rho _1$ and $\rho _2$

For the geometric application we are interested in, we are primarily interested in affine transformation matrices $\mathcal{A}$ that are reasonably close to the unit matrix multiplied by some scalar scaling factor. Therefore, we will assume that both $\rho _1 > 0$ and $\rho _2 > 0$. Additionally, we will for the following parameterizations use the convention to order the singular values such that $\rho _1 > \rho _2$. Then, we obtain

86 $$\begin{aligned} P&= \frac{1}{2} \, (\rho _1 + \rho _2), \end{aligned}$$

87 $$\begin{aligned} Q&= \frac{1}{2} \, (\rho _1 - \rho _2), \end{aligned}$$

which in turn gives

88 $$\begin{aligned} \rho _1&= P + Q, \end{aligned}$$

89 $$\begin{aligned} \rho _2&= P - Q. \end{aligned}$$

#### Relationships for the angular parameters $\alpha$, $\beta$, $\varphi$ and $\psi$

By combining (78) and (79), we additionally get

90 $$\begin{aligned} \frac{A}{T} = \tan (\alpha - \beta ), \end{aligned}$$

and by combining (80) and (81), we get

91 $$\begin{aligned} \frac{S}{C} = \tan (\alpha + \beta ). \end{aligned}$$

Let us next introduce the new variables

92 $$\begin{aligned} \varphi&= \alpha - \beta , \end{aligned}$$

93 $$\begin{aligned} \psi&= \alpha + \beta , \end{aligned}$$

which correspond to the relationships

94 $$\begin{aligned} \alpha&= \frac{\psi + \varphi }{2}, \end{aligned}$$

95 $$\begin{aligned} \beta&= \frac{\psi - \varphi }{2}. \end{aligned}$$

Then, we obtain the solutions

96 $$\begin{aligned} \varphi&= \arctan \left( \frac{A}{T} \right) + n_{\varphi } \, \pi , \end{aligned}$$

97 $$\begin{aligned} \psi&= \arctan \left( \frac{S}{C} \right) + n_{\psi } \, \pi , \end{aligned}$$

for some integers $n_{\varphi }$ and $n_{\psi }$, where we, for the purpose of parameterizing affine transformation matrices $\mathcal{A}$, prefer to choose the principal solutions according to

98 $$\begin{aligned} \varphi&= \operatorname {atan2}(A, T), \end{aligned}$$

99 $$\begin{aligned} \psi&= \operatorname {atan2}(S, C), \end{aligned}$$

where $\operatorname {atan2}(y, x)$ denotes the function that returns the angle between the positive *x*-axis and the vector from the origin to the point (*x*, *y*), with the restriction that this angle assumes values in the interval $]-\pi , \pi ]$.

#### Resulting matrix decompositions

From the above explicit expressions for the angles $\alpha$ and $\beta$, we then obtain that the decomposition (66) can be written as

100 $$\begin{aligned} A&= \mathcal{R}_{\alpha } \, \operatorname {diag}(\rho _1, \rho _2) \, \mathcal{R}_{\beta }^T \nonumber \\&= \mathcal{R}_{\frac{\psi + \varphi }{2}} \, \operatorname {diag}(\rho _1, \rho _2) \, \mathcal{R}_{\frac{\psi - \varphi }{2}}^T \nonumber \\&= \mathcal{R}_{\frac{\psi }{2}} \, \mathcal{R}_{\frac{\varphi }{2}} \, \operatorname {diag}(\rho _1, \rho _2) \, \mathcal{R}_{\frac{\varphi }{2}} \, \mathcal{R}_{\frac{\psi }{2}}^{T}, \end{aligned}$$

which can also be expressed on the more symmetric form

101 $$\begin{aligned} \mathcal{A} = \sqrt{\rho _1 \, \rho _2} \,\, \mathcal{R}_{\frac{\psi }{2}} \, \mathcal{R}_{\frac{\varphi }{2}} \operatorname {diag}\left( \sqrt{\frac{\rho _1}{\rho _2}}, \sqrt{\frac{\rho _2}{\rho _1}}\right) \, \mathcal{R}_{\frac{\varphi }{2}} \, \mathcal{R}_{-\frac{\psi }{2}}. \end{aligned}$$

### Analysis of the proposed decomposition of 2-D affine transformations for special subgroups of affine transformations

In the following, we will compute the parameters in the proposed decomposition of affine transformations according to (101) for four basic classes of image transformations, in terms of uniform scaling transformations, pure rotations and non-uniform scaling transformation.

#### Uniform scaling transformations

For a uniform scaling transformation with spatial scaling factor $S_x > 0$ and with the corresponding spatial scaling transformation matrix

102 $$\begin{aligned} \mathcal{S} = \left( \begin{array}{cc} S_x & 0 \\ 0 & S_x \end{array} \right) , \end{aligned}$$

we have

103 $$\begin{aligned} T&= \frac{a_{11} + a_{22}}{2} = S_x \end{aligned}$$

104 $$\begin{aligned} A&= \frac{a_{21} - a_{12}}{2} = 0, \end{aligned}$$

105 $$\begin{aligned} C&= \frac{a_{11} - a_{22}}{2} = 0, \end{aligned}$$

106 $$\begin{aligned} S&= \frac{a_{12} + a_{21}}{2} = 0, \end{aligned}$$

which then gives

107 $$\begin{aligned} P&= \sqrt{T^2 + A^2} = S_x, \end{aligned}$$

108 $$\begin{aligned} Q&= \sqrt{C^2 + S^2} = 0, \end{aligned}$$

109 $$\begin{aligned} \rho _1&= P + Q = S_x, \end{aligned}$$

110 $$\begin{aligned} \rho _2&= P - Q = S_x, \end{aligned}$$

111 $$\begin{aligned} \varphi&= \operatorname {atan2}\left( A, T \right) = 0, \end{aligned}$$

112 $$\begin{aligned} \psi&= \operatorname {atan2}\left( S, C \right) = \text{ undefined }. \end{aligned}$$

That the angle $\psi$ is undefined here, means that this angle is formally underdetermined, and that any value of $\psi$ will be consistent with the decomposition (101). Without loss of generality, we can therefore make the simplest choice of $\psi = 0$.

#### Pure rotations

For a pure rotation with rotation angle $\gamma$ and the corresponding rotation matrix

113 $$\begin{aligned} \mathcal{R} = \left( \begin{array}{cc} \cos \gamma & -\sin \gamma \\ \sin \gamma & \cos \gamma \end{array} \right) , \end{aligned}$$

we have

114 $$\begin{aligned} T&= \frac{a_{11} + a_{22}}{2} = \cos \gamma , \end{aligned}$$

115 $$\begin{aligned} A&= \frac{a_{21} - a_{12}}{2} = \sin \gamma , \end{aligned}$$

116 $$\begin{aligned} C&= \frac{a_{11} - a_{22}}{2} = 0, \end{aligned}$$

117 $$\begin{aligned} S&= \frac{a_{12} + a_{21}}{2} = 0, \end{aligned}$$

which then gives

118 $$\begin{aligned} P&= \sqrt{T^2 + A^2} = 1, \end{aligned}$$

119 $$\begin{aligned} Q&= \sqrt{C^2 + S^2} = 0, \end{aligned}$$

120 $$\begin{aligned} \rho _1&= P + Q = 1, \end{aligned}$$

121 $$\begin{aligned} \rho _2&= P - Q = 1, \end{aligned}$$

122 $$\begin{aligned} \varphi&= \operatorname {atan2}\left( A, T\right) = \operatorname {atan2}\left( \sin \gamma , \cos \gamma \right) = \gamma \end{aligned}$$

123 $$\begin{aligned} \psi&= \operatorname {atan2}\left( S, C \right) = \text{ undefined }. \end{aligned}$$

Again, since the angle $\psi$ is not formally restricted in the decomposition (101), we make the simplest choice of choosing $\psi = 0$.

#### Non-uniform scaling transformations

For a non-uniform scaling transformation of the form

124 $$\begin{aligned} A&= \mathcal{R}_{\frac{\gamma }{2}} \operatorname {diag}(S_1, S_2) \, \mathcal{R}_{\frac{\gamma }{2}}^T \nonumber \\&= \left( \begin{array}{cc} \cos \frac{\gamma }{2} & -\sin \frac{\gamma }{2} \\ \sin \frac{\gamma }{2} & \cos \frac{\gamma }{2} \end{array} \right) \left( \begin{array}{cc} S_1 & 0 \\ 0 & S_2 \end{array} \right) \left( \begin{array}{cc} \cos \frac{\gamma }{2} & \sin \frac{\gamma }{2} \\ -\sin \frac{\gamma }{2} & \cos \frac{\gamma }{2} \end{array} \right) \end{aligned}$$

with the spatial scaling factors $S_1> S_2 > 0$ and the orientation $\frac{\gamma }{2}$ of the preferred symmetry axis, we do after a straightforward expansion of the matrix product followed by simplifications get that

125 $$\begin{aligned} T&= \frac{a_{11} + a_{22}}{2} = \frac{S_1 + S_2}{2}, \end{aligned}$$

126 $$\begin{aligned} A&= \frac{a_{21} - a_{12}}{2} = 0, \end{aligned}$$

127 $$\begin{aligned} C&= \frac{a_{11} - a_{22}}{2} = \frac{(S_1 - S_2)}{2} \, \cos \gamma , \end{aligned}$$

128 $$\begin{aligned} S&= \frac{a_{12} + a_{21}}{2} = \frac{(S_1 - S_2)}{2} \, \sin \gamma , \end{aligned}$$

which then gives

129 $$\begin{aligned} P&= \sqrt{T^2 + A^2} = \frac{S_1 + S_2}{2}, \end{aligned}$$

130 $$\begin{aligned} Q&= \sqrt{C^2 + S^2} = \frac{|S_1 - S_2|}{2} = \frac{S_1 - S_2}{2}, \end{aligned}$$

131 $$\begin{aligned} \rho _1&= P + Q = S_1, \end{aligned}$$

132 $$\begin{aligned} \rho _2&= P - Q = S_2, \end{aligned}$$

133 $$\begin{aligned} \varphi&= \operatorname {atan2}(A, T) = 0, \end{aligned}$$

134 $$\begin{aligned} \psi&= \operatorname {atan2}\left( S, C \right) = \operatorname {atan2}\left( \sin \gamma , \cos \gamma \right) = \gamma . \end{aligned}$$

In this respect, the proposed sign conventions for the singular values $\rho _1$ and $\rho _2$, as well as the choices for determining the angle $\psi$, correctly recover the parameters in the original non-uniform transformation of the form (124).

#### Pure skewing transformations

Consider a pure skewing transformation with skewing angle $\gamma$ of the form

135 $$\begin{aligned} x'&= x + y \, \tan \gamma , \end{aligned}$$

136 $$\begin{aligned} y'&= y, \end{aligned}$$

that is with an affine transformation matrix of the form

137 $$\begin{aligned} \mathcal{A} = \left( \begin{array}{cc} 1 & \tan \gamma \\ 0 & 1 \end{array} \right) , \end{aligned}$$

we have

138 $$\begin{aligned} T&= \frac{a_{11} + a_{22}}{2} = 1, \end{aligned}$$

139 $$\begin{aligned} A&= \frac{a_{21} - a_{12}}{2} = - \frac{\tan \gamma }{2}, \end{aligned}$$

140 $$\begin{aligned} C&= \frac{a_{11} - a_{22}}{2} = 0, \end{aligned}$$

141 $$\begin{aligned} S&= \frac{a_{12} + a_{21}}{2} = - \frac{\tan \gamma }{2}, \end{aligned}$$

which then gives

142 $$\begin{aligned} P&= \sqrt{T^2 + A^2} = \sqrt{1 + \frac{\tan ^2 \gamma }{4}}, \end{aligned}$$

143 $$\begin{aligned} Q&= \sqrt{C^2 + S^2} = \frac{| \tan \gamma |}{2}, \end{aligned}$$

144 $$\begin{aligned} \rho _1&= P + Q = \sqrt{1 + \frac{\tan ^2 \gamma }{4}} + \frac{| \tan \gamma |}{2}, \end{aligned}$$

145 $$\begin{aligned} \rho _2&= P - Q = \sqrt{1 + \frac{\tan ^2 \gamma }{4}} - \frac{| \tan \gamma |}{2}, \end{aligned}$$

146 $$\begin{aligned} \varphi&= \operatorname {atan2}\left( A, T \right) = \operatorname {atan2}\left( - \frac{\tan \gamma }{2}, 1 \right) \nonumber \\&= \arctan \left( - \frac{\tan \gamma }{2} \right) , \end{aligned}$$

147 $$\begin{aligned} \psi&= \operatorname {atan2}\left( S, C \right) = \operatorname {atan2}\left( - \frac{\tan \gamma }{2}, 0 \right) = - \frac{\pi }{2} \, \operatorname {sign}\gamma . \end{aligned}$$

In other words, according to the proposed decomposition (101) of affine image transformations, a pure skewing transformation is regarded as a more complex image transformation, that corresponds to (i) first rotating the input image to a preferred symmetry orientation in the image plane by the angle $-\frac{\psi }{2}$, then (ii) rotating the image by the angle $\frac{\varphi }{2}$ before (iii) performing a pure non-uniform scaling transformation with the spatial scaling factors $\rho _1$ and $\rho _2$, after which the image is again (iv] rotated by the same angle $\frac{\varphi }{2}$, before finally being (vi) rotated back from the preferred symmetry orientation.

## Footnotes

1 Note that in a general singular value decomposition of an arbitrary affine transformation matrix $\mathcal{A}$ of the form $\mathcal{A} = U \, \varSigma \, V^T$, the matrices *U* and *V* are only restricted to be unitary matrices, not necessarily rotation matrices.

2 The tilt direction is the direction where the depth along a smooth surface varies most strongly.

3 Note, however, that the treatment in this paper goes conceptually further compared to the earlier treatments in Lindeberg (2023, 2025a), by explicitly addressing a variability in receptive field shapes along the fourth degree of freedom in Section 7.4, which was not addressed in Lindeberg (2023, 2025a).
